# Reduced Expression of VAMP8 in Lacrimal Gland Affected by Chronic Graft-versus-Host Disease

**DOI:** 10.1155/2017/1639012

**Published:** 2017-09-30

**Authors:** Masaki Fukui, Yoko Ogawa, Shin Mukai, Mizuka Kamoi, Teru Asato, Yutaka Kawakami, Kazuo Tsubota

**Affiliations:** ^1^Department of Ophthalmology, Keio University School of Medicine, Tokyo, Japan; ^2^Institute for Advanced Medical Research, Keio University School of Medicine, Tokyo, Japan

## Abstract

**Purpose:**

To investigate whether the SNARE protein vesicle-associated membrane protein 8 (VAMP8) was implicated in the development of chronic ocular graft-versus-host disease (GVHD).

**Methods:**

Firstly, the chronic GVHD (cGVHD) and Sjögren's syndrome (SS)-impaired lacrimal gland (LG) tissue sections from humans for diagnostic purpose were evaluated for VAMP8 expression by histopathology and immunohistochemistry. Next, serial changes of tear secretion and VAMP8 expression at both protein and mRNA level of LG in an animal cGVHD model compared with the syngeneic control.

**Results:**

Decreased VAMP 8 expression in the cGVHD-affected human LG was detected in comparison with SS-affected LG. Tear secretion in the murine cGVHD model was significantly reduced compared with that in the syngeneic controls 8 weeks after BMT. Protein expression of VAMP8 in the cGVHD-affected LG in murine cGVHD was decreased in comparison with that in the controls. Gene expression of VAMP8 in the cGVHD-affected murine LG was significantly less than that in the syngeneic control 3 weeks after BMT.

**Conclusions:**

Our results suggested that expression of VAMP8 in the cGVHD-affected LG was decreased and accordingly tear secretion in cGVHD was reduced. Collectively, the reduction of VAMP8 expression in the cGVHD-affected LG can be involved in the pathogenic processes of cGVHD-induced dry eye disease.

## 1. Introduction

Chronic graft-versus-host disease (cGVHD) is one of the complications that occur after hematopoietic stem cell transplantation (HSCT). cGVHD arises from immune response of donor grafts to recipient cells and/or tissues, and cGVHD-susceptible organs are the eyes, mouth, lung, skin, small and large intestine, and liver [1–4]. Exocrine glands and mucosal epithelia are one of the cGVHD target tissues. Exocrine glands including the lacrimal glands and ocular surface mucosal epithelia such as the conjunctiva are highly vulnerable to cGVHD. Damaged microvilli and dysfunctional production of gel-forming and/or membrane-spanning mucin are observed in the cGVHD-impaired human conjunctiva [5]. In addition, reduced tear secretion and severely inflamed lacrimal gland epithelia are reported in cGVHD-affected patients [6]. Since dry eye disease is the most common disorder elicited by ocular GVHD, further elucidation on the pathogenic process of cGVHD-related dry eye disease is awaited.

VAMP8 is one of the soluble N-ethylmaleimide-sensitive factor attachment protein receptor (SNARE) proteins, which are required when exocrine glands release secretory granules [7]. VAMP8 has been reported to play a role in regulating exocytosis in the exocrine system. In this process, VAMP8 serves as a v-SNARE of zymogen granules [8]. Therefore, it was envisioned that VAMP8 could be related to exocytosis of secretory vesicles in lacrimal gland epithelia. We reported that VAMP8 was abnormally expressed in the human lacrimal gland impaired by Sjögren's syndrome (SS) [9].

However, the relevance between SNARE proteins and cGVHD-related dry eye disease has yet to be elucidated. In this study, we investigated whether the SNARE protein VAMP8 was implicated in the development of lacrimal gland cGVHD in humans and mice.

## 2. Materials and Methods

### 2.1. Patients

For human study, written informed consent was obtained in advance from all patients. Institutional Review Board at Keio University School of Medicine approved for this study including all experimental methods for humans in accordance with the tenets of the Declaration of Helsinki (number 20090277). Biopsies were conducted for the diagnostic purpose of patients with cGVHD or SS. The remaining lacrimal gland and conjunctiva for the diagnosis were analyzed for this study. We examined lacrimal gland and conjunctival specimens from three patients who had received allogeneic HSCT and in whom dry eye disease developed later (cases 1–3). Lacrimal gland and conjunctival specimens from three patients with SS were examined as control samples (cases 4–6) (Table 1). All subjects were recruited from patients attending the dry eye outpatient clinic at Keio University Hospital. Dry eye disease was diagnosed as a disorder of the tear film caused by tear deficiency or excessive tear evaporation, which causes damage to the ocular surface, with symptoms of ocular discomfort [10].

### 2.2. Lacrimal Gland Biopsy

We followed the methods of Ogawa et al. published in 2001 [11]. The number of human samples was small, because lacrimal gland biopsy is not performed routinely for diagnostic purposes.

### 2.3. Light Microscopic Examination

All lacrimal gland and conjunctival specimens were immediately fixed in 10% neutralized buffered formalin, embedded in paraffin wax, and processed according to conventional histologic techniques, including Hematoxylin-Eosin staining [11].

### 2.4. Animal Experiments

All animal experiments were conducted in accordance with the ARVO Statement for the Use of Animals in Ophthalmic and Vision Research following the approval of the protocol number 09152 by the Ethics Committee on Animal Research of the Keio University School of Medicine. We followed the ARRIVE guidelines for reporting *in vivo* experiments in animal research [12]. We followed the methods of Zhang et al. published in 2013 [13].

### 2.5. Materials

For cell surface antigen, CD45 rat monoclonal antibody (catalog number 14-0451-82; clone 30-F11) was from eBioscience/Thermo Fisher Scientific (San Diego, CA, USA). VAMP8 rabbit monoclonal antibody (catalog number ab76021; clone EP2629Y) for human and mouse was from Abcam (Cambridge, UK). Mouse anti-human E-cadherin monoclonal antibody (catalog number SC-21791; clone 67A4) was from Santa Cruz Biotechnology (Dallas, TX, USA), and rabbit anti-mouse E-cadherin monoclonal antibody (catalog number 3195S; clone 24E10) was from Cell Signaling Technology (Danvers, MA, USA). Isotype-matched antibodies including rat IgG2b, k for CD45, mouse Ig G1 antibody or rabbit Ig G antibody for E-cadherin, and rabbit IgG antibody for VMAP8 were prepared for negative controls. 10% goat serum (catalog number 50062Z) was purchased from Life Technologies (Carlsbad, CA, USA). Antigen retrieval solution (Target Retrieval Solution, catalog number S169984) was purchased from Dako/Agilent (Santa Clara, CA, USA).

An RNeasy mini kit and a ReverTra Ace with genomic remover qPCR RT Kit were purchased from Qiagen (Hilden, Germany). The primers for VAMP8 including glyceraldehyde-3-phosphate dehydrogenase (GAPDH) for mRNA expression analysis by TaqMan real-time PCR were purchased from Applied Biosystems (Foster City, CA, USA). We used the following mouse TaqMan probe for quantitative real-time PCR: Mm00450314_m1 for VAMP8 and Mm99999915_g1 for GAPDH.

### 2.6. B10.D2 → BALB/c (H-2d) Murine Model of cGVHD

Eight-week-old male B10.D2 and female BALB/c mice (H-2d, Sankyo Laboratory Inc., Tokyo, Japan) were utilized as bone marrow transplantation (BMT) donors and recipients, respectively, to produce cGVHD, using a previously reported method [13]. Briefly, female recipient mice were lethally irradiated with 700 cGy of X-ray (X-ray device; MRB-1520R-3; Hitachi Medical Co., Tokyo, Japan). Approximately 6 hours later, they were injected in the tail vein with male donor bone marrow (1 × 10^6^/mouse) and spleen cells (2 × 10^6^/mouse) suspended in 200 *μ*L of RPMI medium 1640 (Life Technologies). Another group of female BALB/c recipient mice was lethally irradiated with 700 cGy of X-ray and then received male BALB/c spleen and bone marrow cells, as syngeneic BMT control animals.

### 2.7. Immunohistochemical Experiments on Lacrimal Gland and Conjunctival Samples

Paraffin-embedded sections were used for immunohistochemistry as previously described. Briefly, consecutive 6 *μ*m thick paraffin-embedded sections were deparaffinized, rehydrated, and washed with PBS. Antigen unmasking was performed by microwaving for 10 minutes with 11 minutes preheating for E-cadherin and VAMP8. The sections were then blocked with 10% goat serum for 30 minutes and then incubated overnight at 4°C with the following primary antibodies: CD45 rat anti-mouse antibody for single staining, mouse anti-human E-cadherin, and rabbit anti-human VAMP8 for double staining. After being washed with PBS, the sections were treated with an Alexa Fluor 488-conjugated goat anti-rat secondary antibody (Thermo Fisher Scientific, Rockford, IL, USA) for CD45 single staining and an Alexa Fluor 488-conjugated goat anti-mouse secondary antibody and an Alexa Fluor 568-conjugated goat anti-rabbit secondary antibody (Thermo Fisher Scientific) for double staining for 45 minutes and washed with PBS. Nuclei were counterstained with 4′,6-diamidino-2′-phenylindole dihydrochloride (DAPI) (Life Technologies). The tissue sections used for fluorescent staining were mounted on glass slides and examined with a confocal microscope (LSM700-ZEN 2009, Carl Zeiss MicroImaging GmbH, Jena, Germany). To assess the histological architecture and staining, all of the acquired images were reviewed twice each by two independent observers (M. F. and Y. O.) who were blinded to the source of the samples.

### 2.8. Transmission Electron Microscopy (TEM)

A portion of lacrimal gland was fixed with 2.5% glutaraldehyde and subjected to an examination by TEM as described previously [11]. One-micrometer thick sections were stained with methylene blue and thin sections were made with a diamond knife. The sections were collected on mesh grids, stained with uranyl acetate and lead citrate, and examined under an electron microscope (1230 EXII; JOEL, Tokyo, Japan). All photographs were taken with a bio scan camera (Gatan bio scan camera model 792, Tokyo, Japan).

### 2.9. Measurement of Tear Secretion

The mice were anesthetized by intraperitoneal injection of 5 mg/mL sodium pentobarbital (Dainihon Sumitomo Seiyaku Co. Ltd., Tokyo, Japan). Tear production was stimulated by intraperitoneal injection of 3 mg/kg pilocarpine (Santen, Osaka, Japan) at 1 minute after the anesthesia. Tear fluids were collected for 15 minutes, and the volume was calculated every 5 minutes during the 15 minutes duration using 0.5 *μ*L capillary microglass tubes (Microcaps, Drummond Scientific Company, PA, USA). After the measurement, the mice were sacrificed, and the lacrimal gland was removed. Then, the lacrimal gland weights were measured, and the mean values were calculated to obtain the average lacrimal gland weight of the mice. The tear secretion volume was adjusted for the weight of each lacrimal gland [14].

### 2.10. Quantitative Real-Time Polymerase Chain Reaction (PCR)

Total RNA was extracted from lacrimal gland using an RNeasy mini kit (Qiagen), and cDNA synthesis was performed using a ReverTra Ace qPCR RT Kit (Toyobo Co. Ltd., Osaka, Japan). The primers for mRNA expression analysis by TaqMan real-time polymerase chain reaction (PCR) were purchased from Applied Biosystems for the housekeeping gene glyceraldehyde-3-phosphate dehydrogenase (GAPDH) and VAMP8. Quantitative real-time PCR was performed using the Step One Plus system (Applied Biosystems). All data were analyzed with the 2^−ΔΔCT^ method, and the mRNA of GAPDH was used as the internal standard.

### 2.11. Statistical Analyses

All results are expressed as the mean ± SD. The data were subjected to statistical analyses (unpaired Student's *t*-test, the R statistical package, R version 3.1.3 (R Core Team (2015)), R: a language and environment for statistical computing, and R Foundation for Statistical Computing, Vienna, Austria, http://www.R-project.org/.) to determine differences between the allogeneic and syngeneic groups. ImageJ (available at: http://www.imagescience.org/meijering/software) [15] was used for mean gray scale of immunostaining of VAMP8 to determine differences between the allogeneic and syngeneic groups. Differences were considered to be statistically significant at *p* < 0.05.

## 3. Results

### 3.1. Histopathology of Human Lacrimal Gland and Conjunctiva

**Figure 1:**
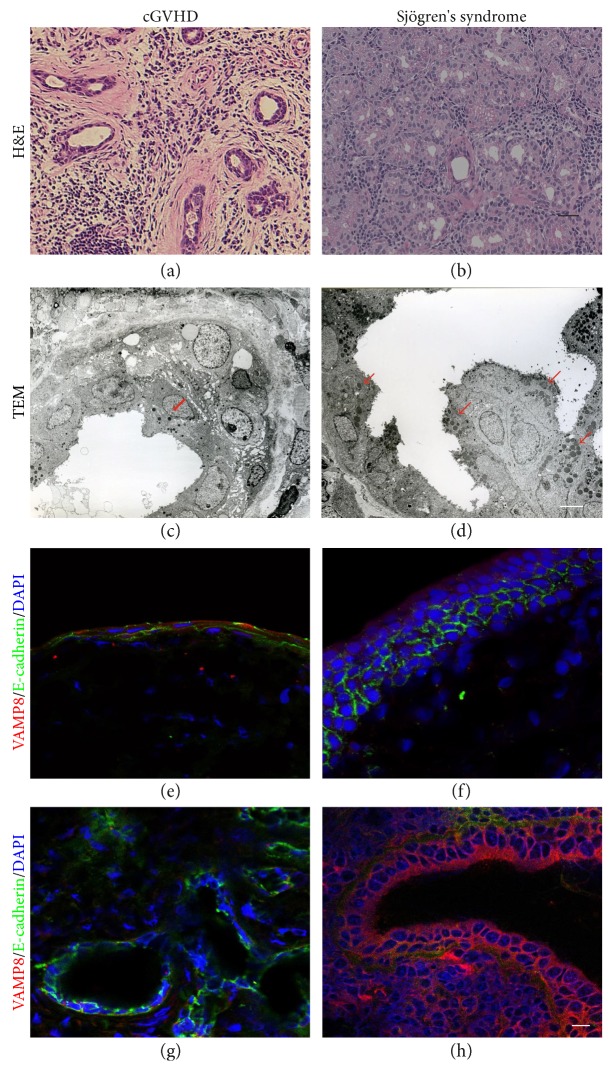
Pathological findings and confocal microscopic images of human cGVHD-affected lacrimal gland and conjunctival epithelia. Hematoxylin & eosin (H&E) staining. GVHD, case 3 (a); Sjögren's syndrome (SS), case 2 (b). Transmission electron microscopic (TEM) findings. GVHD, case 2 (c); SS, case 1 (d). (c, d) Red arrows, secretory vesicle immunostaining for VAMP8 and E-cadherin expression in conjunctival epithelia (e, f) and lacrimal gland (g, h) from cGVHD (case 3) (e, g) and SS (case 2) (f, h) patient. VAMP8 is shown in red, E-cadherin in green, and 4′,6-diamidino-2-phenylindole (DAPI) in blue. Scale bar = 50 *μ*m (a), 100 *μ*m (b), 4 *μ*m (c, d), 25 *μ*m (e, f), and 20 *μ*m (g, h).

The cGVHD-affected tissues had several histological features in common with the SS-affected ones [6]. However, the lacrimal gland tissues in patients with cGVHD were distinct from those in patients with SS in the following aspects. Excessive fibrosis and numerous inflammatory cells were observed in the cGVHD-impaired lacrimal gland stroma, and accordingly, the acinar cells were thin and atrophic (Figure 1(a)). On the other hand, the remaining acinar cells in the SS-affected lacrimal gland seemed to be virtually intact, although a large number of inflammatory cells infiltrated into the tissues (Figure 1(b)).

### 3.2. Transmission Electron Microscopy of Human Lacrimal Gland

To examine the status of secretory vesicles in the lacrimal gland epithelia, we investigated three human cGVHD and two human SS samples with an electron microscope (Figures 1(c) and 1(d)). The number of secretory vesicles in the cGVHD-affected lacrimal gland epithelia was less (Figure 1(c)) than that in its SS-affected counterparts (Figure 1(d)).

### 3.3. Immunohistochemistry of Human Conjunctiva and Lacrimal Gland

We examined VAMP8 expression in the conjunctiva and lacrimal gland epithelia (Figures 1(e), 1(f), 1(g), and 1(h)). To discriminate each epithelium in the conjunctiva and lacrimal gland, we conducted double staining for VAMP8 and the intercellular adhesion molecule E-cadherin. A typical histopathological finding in the conjunctival epithelia from patients with cGVHD was that it was much thinner (Figure 1(e)) compared with those from SS patients (Figure 1(f)). VAMP8 expression was diminished in the cGVHD-affected conjunctival epithelia (Figure 1(e)) in comparison with the SS-affected counterparts (Figure 1(f)). The thinning of the epithelia and the decrease in VAMP8 expression in the epithelia were also observed in the cGVHD-impaired lacrimal gland (Figure 1(g)). However, these phenomena were not obvious in the SS-impaired remaining epithelia (Figure 1(h)), although they seemed to be markedly inflamed. These results suggested that (1) the fusion of secretory vesicles from cytoplasm and apical membrane in the lacrimal gland and conjunctiva and (2) the maturation of secretory vesicles were vulnerable to cGVHD.

### 3.4. Murine Model of cGVHD

To examine whether VAMP8 expression was decreased in lacrimal gland cGVHD epithelia, we used an established animal model of cGVHD [13]. In this mouse model, the MHC is identical and the minor histocompatibility antigens are mismatched. Hence, this murine model of cGVHD closely mimics clinical findings in patients suffering from cGVHD-related dry eye disease.

### 3.5. Histopathology of Murine Lacrimal Gland

**Figure 2:**
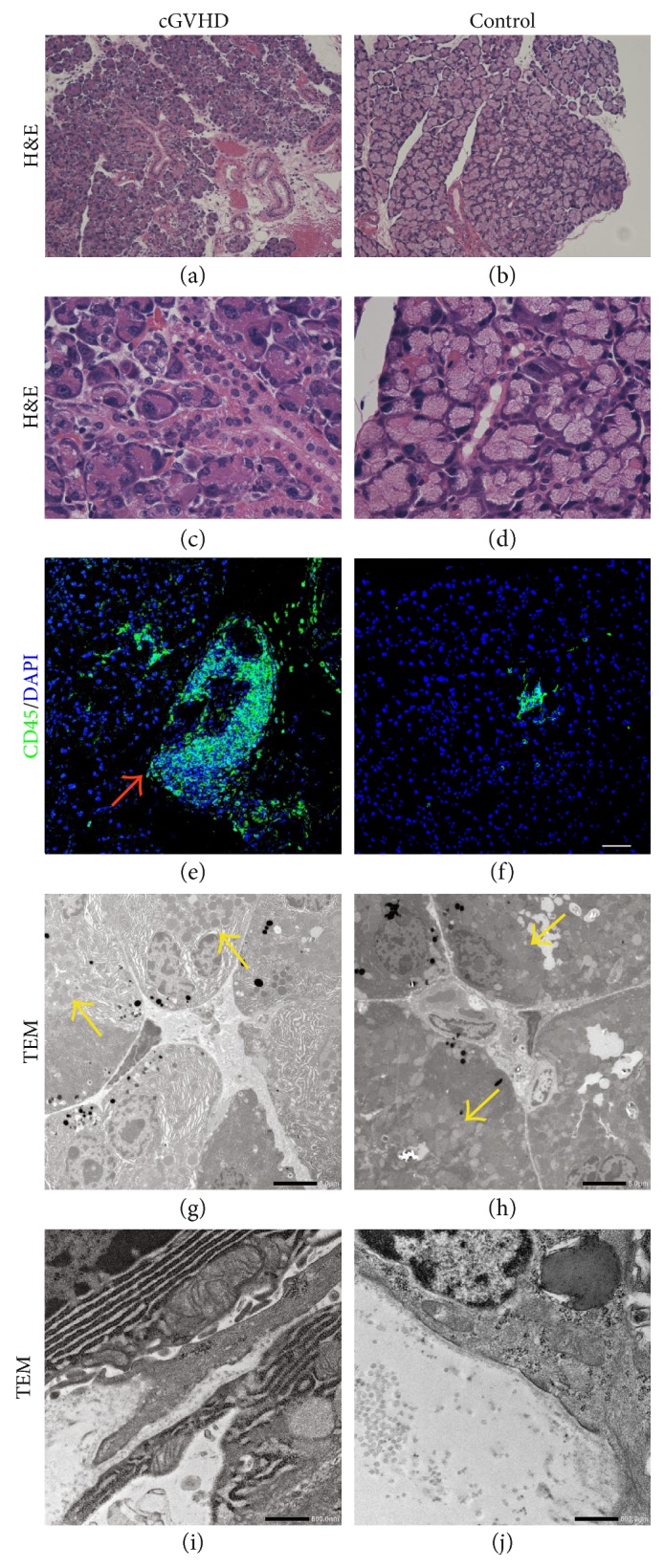
Characteristics histopathological findings of lacrimal gland in a mouse model of cGVHD. Hematoxylin & eosin (H&E) staining for lacrimal gland tissue sections from GVHD-affected mouse model (a, c) and syngeneic control (b, d). Immunostaining for CD45^+^ cells (in green and red arrow) and cell nuclei (stained with 4′,6-diamidino-2-phenylindole (DAPI) in blue) in the lacrimal gland collected from the GVHD-impaired mice (e) and syngeneic controls (f). Transmission electron microscopic (TEM) findings of the lacrimal gland in a mouse model of cGVHD (g, i) and the syngeneic controls (h, j). (g, h) Yellow arrows, secretory vesicles. Scale bar = 50 *μ*m (a, b), 25 *μ*m (c, d), 50 *μ*m (e, f), 5 *μ*m (g, h), and 0.5 *μ*m (i, j). cGVHD mice, *n* = 10; syngeneic control mice, *n* = 10. Representative figures from three independent experiments.

H&E images of the cGVHD-impaired murine lacrimal gland showed inflammatory cell infiltration into the stroma and decreased secretory granules in the epithelia (Figures 2(a), 2(b), 2(c), and 2(d)). Immunostaining revealed the infiltration of CD45^+^ cells in the lacrimal gland affected by cGVHD (Figure 2(e)) in comparison to the syngeneic controls (Figure 2(f)).

### 3.6. Transmission Electron Microscopy of Murine Lacrimal Gland

The lacrimal gland epithelial cells in the mice with cGVHD had less secretory granules, more inflammatory cells and fibrotic in the stroma (Figure 2(g)) than those in syngeneic control mice (Figure 2(h)). The basal lamina was attenuated in the cGVHD mice (Figure 2(i)), whereas this finding was not observed in the syngeneic controls (Figure 2(j)). These outcomes indicated that this animal model recapitulated the histological findings of human cGVHD-affected lacrimal gland epithelia.

### 3.7. Immunohistochemistry of Murine Lacrimal Gland

**Figure 3:**
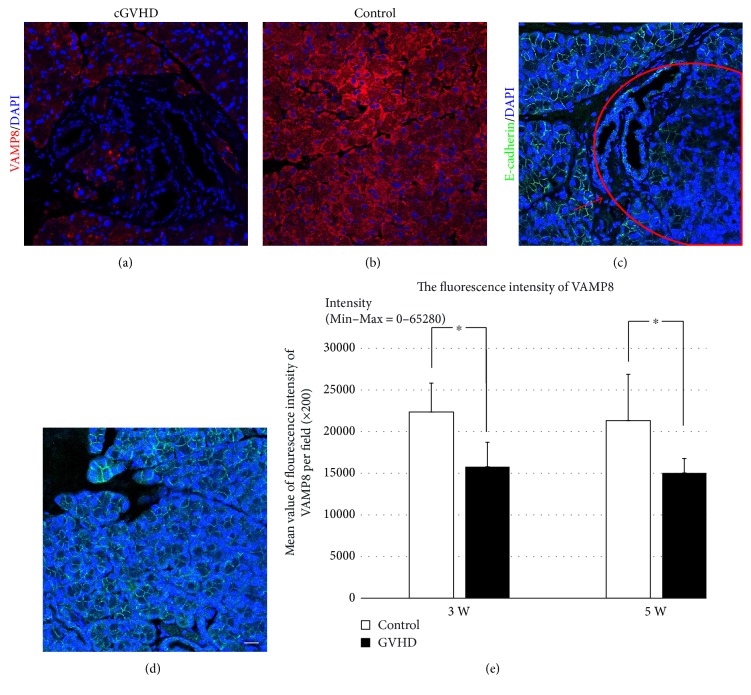
VAMP8 and E-cadherin expression in cGVHD-impaired murine lacrimal gland. VAMP8 expression in the lacrimal gland epithelia affected by cGVHD (a) and in the syngeneic control subjects (b). E-cadherin expression in the lacrimal gland epithelia in the GVHD-affected mice ((c), red boxed area, reduction of E-cadherin expression) and syngeneic control (d). VAMP8 is shown in red, E-cadherin in green, and 4′,6-diamidino-2-phenylindole (DAPI) in blue. Scale bar = 25 *μ*m (a, b, c, and d). The vertical axis shows the average of fluorescence intensity of VAMP8 measured by ImageJ (e). The intensity of VAMP8 in the syngeneic control and GVHD-affected mice at the 3 and 8 weeks after bone marrow transplantation. *N* = 4–5. Bar = mean ± SD. ^∗^*p* < 0.05, *p* = 0.012 at 3 weeks, *p* = 0.042 at 8 weeks. cGVHD mice, *n* = 10; syngeneic control mice, *n* = 10. The experiments were repeated three times.

Our next attempt was to investigate VAMP8 and E-cadherin expression in the murine lacrimal gland epithelia affected by cGVHD (Figures 3(a), 3(b), 3(c), and 3(d)). To attain this goal, we performed immunofluorescent staining for E-cadherin and VAMP8 in lacrimal gland tissue sections from the cGVHD mouse model. The results indicated that VAMP8 expression in the cGVHD-affected lacrimal gland epithelia was significantly repressed (Figures 3(a) and 3(e)) compared with that in the control samples (Figures 3(b) and 3(e)). Statistical analysis of fluorescence intensity of VAMP8 measured by ImageJ showed that VAMP8 expression in the cGVHD-affected lacrimal gland epithelia was significantly decreased compared with that in the control samples (*p* < 0.05, *p* = 0.012 at 3 weeks; *p* = 0.042 at 8 weeks) (Figure 3(e)). Similarly, E-cadherin expression in the cGVHD-impaired lacrimal gland epithelia (Figure 3(c)) was vastly lower compared with that in the syngeneic control subjects (Figure 3(d)). As with the human cGVHD subjects, the fusion of secretory vesicles from cytoplasma and apical membrane in the murine lacrimal gland and the maturation of secretory vesicles can be susceptible to cGVHD.

### 3.8. Tear Secretion in Wild-Type, cGVHD-Affected, and Syngeneic Control Mice

**Figure 4:**
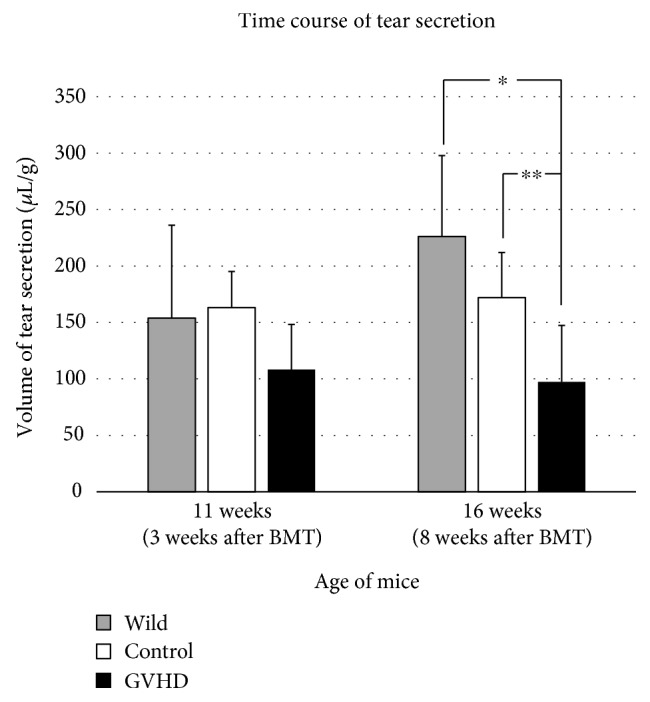
Comparison of tear secretion in wild-type, syngeneic control, and GVHD-affected mice. The vertical axis shows the volume of secreted tears. The volume of tear secretion is adjusted for weight of each lacrimal gland (*μ*L/g). Tear secretion in the wild-type (gray bars), syngeneic control (white bars), and GVHD-affected mice (black bars) at the age of 11 (3 weeks after bone marrow transplantation (BMT) for the syngeneic control and GVHD-affected mice) and 16 weeks (8 weeks after BMT for the syngeneic control and GVHD-affected mice). *n* = 2–5. Bar = mean ± SD. ^∗^*p* = 0.011, ^∗∗^*p* = 0.073. The experiments were performed three times.

We measured the tear secretion in wild-type, cGVHD-affected, and syngeneic control mice (Figure 4). Eight-week-old mice underwent allogenic or syngeneic BMT, and their tear production was measured 3 and 8 weeks after transplantation. The wild-type mice were as old as those subjected to BMT. The tear secretion in the syngeneic control mice 3 and 8 weeks after BMT is no difference of that of wild-type mice age at 11 and 16 weeks, respectively (*p* > 0.05). At both 3 and 8 weeks after BMT, the tear secretion of cGVHD mice was less than that of control mice. Notably, the tear secretion of cGVHD mice was significantly reduced 8 weeks after BMT compared with that of the syngeneic control mice (*p* < 0.05).

### 3.9. Gene Expression of VAMP8

**Figure 5:**
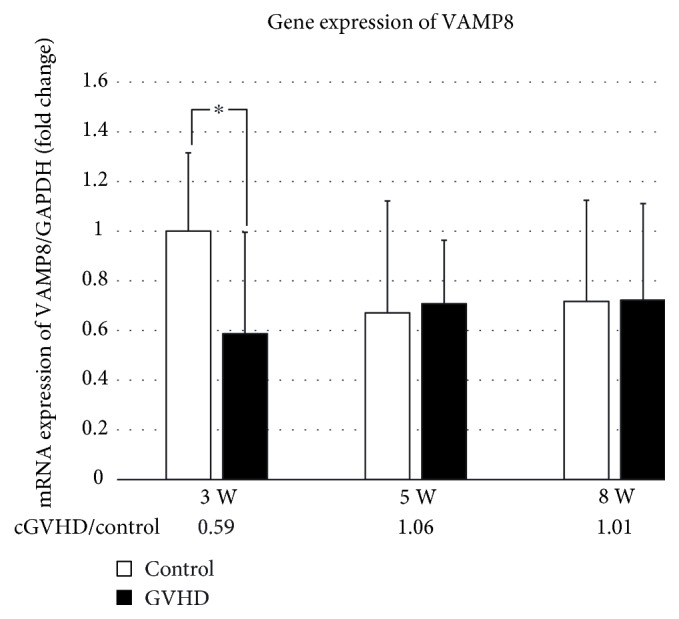
Time course of changes in VAMP8 gene expressions in the lacrimal gland of murine model of cGVHD. The organ was collected from the mice which underwent allogeneic or syngeneic bone marrow transplantation (BMT). The transcript levels of VAMP8 were measured by quantitative real-time PCR 3, 5, and 8 weeks after BMT. (Allogeneic group: *N* = 8 − 9; syngeneic control group: *N* = 8). In this analysis, GAPDH was used as an internal control. Bar = mean ± SD. ^∗^*p* = 0.036. Data from three independent experiments.

As the immunohistochemistrical results suggested the reduction of VAMP, we aimed to elucidate the gene expression of VAMP8 in the lacrimal gland by quantitative real-time PCR (Figure 5). Any time the gene expression of VAMP8 was measured, the results were consistent. The gene expression of VAMP8 in the cGVHD-impaired lacrimal gland was 0.59, 1.06, and 1.01 times higher compared to that of the syngeneic control mice 3, 5, and 8 weeks after BMT, respectively. At 3 weeks after BMT, the gene expression of VAMP8 in the lacrimal gland of cGVHD mice was significantly lower than that of the control mice (*p* < 0.05).

## 4. Discussion

In this study, we found that the cGVHD-induced reduction of VAMP8 was relevant to the reduced expression of E-cadherin. The serial measurement of tear volume in mice with cGVHD revealed that the tear production was gradually declined. Interestingly, the reduction of gene expression of VAMP8 preceded the decrease in tear secretion. Those results were indicative of some relationship between the decrease in tear secretion and the reduction of VAMP8 expression and in lacrimal gland from humans and mice affected by cGVHD.

Histopathological images of the conjunctiva and lacrimal gland from patients with cGVHD showed marked infiltration of inflammatory mononuclear cells and the thinning of the damaged epithelial cells. It is conceivable that these changes can be related to dry eye disease caused by cGVHD. These findings suggested that damaged epithelial cells in the lacrimal gland and conjunctiva impaired by cGVHD could be associated with the decrease in tear secretion.

We next examined the protein expression of VAMP8 and E-cadherin in conjunctival and lacrimal gland epithelia affected by cGVHD. Immunofluorescence images of the conjunctiva and lacrimal gland from patients with cGVHD revealed the impaired expression of E-cadherin and VAMP8 in contrast to samples from patients with SS. The reduction of E-cadherin expression led us to a hypothesis that the damaged conjunctival and lacrimal gland epithelia could cause EMT. The decrease in E-cadherin has also been reported to induce rearrangement of cytoskeleton in the cytoplasm of damaged epithelia [16–18]. In addition, the reduction of VAMP8 expression can be associated with the reduction of tear secretion, possibly because the secretory vesicles are not adequately translocated to the apical membrane of the secretory site [8]. These results suggest the relationship between the damage of epithelial cells and the decrease in tear secretion. As demonstrated by our investigation, the reduction of VAMP8 and E-cadherin was observed shortly after dry eye disease was induced in an established mouse model of cGVHD. In addition, we confirmed that histological and immunohistochemical findings of samples from patients with cGVHD were reminiscent of those from mice.

We chose this animal model because it is well recognized as an established model of cGVHD and because it reflects the inflammation and fibrosis in target organs of cGVHD [19]. We have published several studies on lacrimal gland cGVHD in humans [11, 18] and mice [20, 21] and have observed that the lacrimal gland pathology of this animal model is quite similar to that of humans [11, 18]. Dry eye disease is a major problem for patients suffering from cGVHD after HSCT. Thus, we subsequently monitored changes of tear secretion volume in a mouse model of cGVHD for a certain period of time. As reported previously, excessive inflammation in the lacrimal gland lesion in this animal model was seen [13]. Our study showed that 3 weeks after BMT, the reduction of tear secretion in the cGVHD-affected mice and the increase in CD45^+^ inflammatory cell infiltration into the murine lacrimal gland. These results suggested that tear production in this mouse model of cGVHD started to be declined approximately 21 days after BMT which is reported to be the onset of cGVHD in this animal model [13], because of dry eye disease elicited by cGVHD. The findings considerably resemble those of cGVHD-related dry eye disease in humans. We used intraperitoneal anesthesia and subsequent intraperitoneal pilocarpine. Pilocarpine acts as a nonselective muscarinic receptor agonist, and it stimulates the lacrimal gland to secrete tears. In animal experiments, pilocarpine stimulation was used under anesthesia [14, 22, 23]. We used pentobarbital for intraperitoneal anesthesia. Pilocarpine stimulation was used for observing the maximum tear secretion in GVHD mice, which was always compared with similarly treated syngeneic control mice. Therefore, it is likely that we could eliminate the influence of intraperitoneal anesthesia to tear secretion by using syngeneic control mice.

Next, we examined the gene expression of VAMP8 in the murine lacrimal gland with or without cGVHD. The mRNA level of VAMP8 in the cGVHD-affected lacrimal gland was lower than that in the syngeneic controls 3 weeks after BMT. Moreover, we investigated the time course of changes in expression of VAMP8 in an organ affected by cGVHD, and there is no literature precedent with focused on this aspect. As confirmed by our results, the lacrimal gland from the cGVHD-impaired mice showed the lower mRNA level of VAMP8 than that from the control subjects at early onset of cGVHD in this animal model. These data suggest that the reduction of VAMP8 expression was associated with the onset of cGVHD in the lacrimal gland. In addition, tear secretion was also reduced after the onset of cGVHD in the animal model. These findings suggested that the decrease in VAMP8 expression could contribute to the reduction of tear secretion at the onset of cGVHD-related dry eye disease. VAMP8 is involved in the fusion of secretory granules and cellular membranes [24, 25]. One of the tear components is also secreted from secretory vesicles in the lacrimal gland, and the tear component production is controlled partially by VAMP8 [9, 26]. Our investigation of tear secretion showed that the volume of tears in cGVHD-affected mice was lower than that in wild-type and syngeneic control mice 3 and 8 weeks after BMT, especially significantly lower at 8 weeks after BMT. These results suggest that the decreased gene expression of VAMP8 at 3 weeks after BMT influence a significant reduction in tear secretion at 8 weeks.

In the conjunctiva and lacrimal gland affected by cGVHD, the expression of E-cadherin was reduced and the normal-shaped epithelia were transformed to spindle-shaped ones. Our previous paper demonstrates that EMT contributes to fibrosis in lacrimal gland and conjunctiva in cGVHD patients [18]. As suggested by this study, EMT can affect the reduction of VAMP8 expression, which might be accompanied by the decrease in one of the serous component of tear production. Continuing investigation into the relationship between VAMP8 expression and EMT-related molecules is required to gain greater insights into the molecular process of these phenomena.

The lethal irradiation of this animal model could independently affect tear secretion and/or VAMP8 expression. However, in our study, the tear secretion of syngeneic control mice was not significantly different from that of wild-type mice (Figure 4), even though these syngeneic control mice were irradiated. Therefore, we think that the lethal irradiation did not affect tear secretion.

One limitation in this study is that variability of the disease stages between the enrolled human patients could have affected the results (Table 1). For example, one of the three cGVHD patients (case 3) showed mild dry eye in the clinical examination, but analysis revealed that inflammation and periductal fibrosis already existed in the lacrimal gland (Figure 1). The second limitation is the small number of human samples.

## 5. Conclusion

In summary, this study has paved the way to elucidate the pathogenic process of cGVHD-related dry eye disease. At the onset of cGVHD, the lacrimal gland and conjunctiva are inflamed, and acinar epithelial cells in the lacrimal gland and conjunctival epithelia are damaged and thinning. Subsequently, the reduction of VAMP8 expression at the mRNA and protein levels can result in dysfunctional tear production.

Hence, elucidating a functional role of VAMP8 can be a powerful tool to better understand the underlying mechanism of chronic ocular GVHD, and creating novel methods to promote the production of VAMP8 will be beneficial for the treatment of cGVHD in medical setting.

## Figures and Tables

**Table 1:** Patient characteristics in this study.

Cases	Age	Gender	Severity of dry eye	Systemic complications
GVHD1	24	Female	Severe	Oral, liver GVHD
GVHD2	47	Female	Mild	Oral, liver GVHD
GVHD3	40	Female	Mild	—
SS1	61	Female	Severe	—
SS2	34	Female	Severe	Rheumatoid arthritis
SS3	57	Female	Mild	—
